# Application of the matched nested case-control design to the secondary analysis of trial data

**DOI:** 10.1186/s12874-020-01007-w

**Published:** 2020-05-14

**Authors:** Christopher Partlett, Nigel J. Hall, Alison Leaf, Edmund Juszczak, Louise Linsell

**Affiliations:** 1 Nottingham Clinical Trials Unit, University of Nottingham, Nottingham, UK; 2 National Perinatal Epidemiology Unit, Nuffield Department of Population Health, University of Oxford, Oxford, UK; 3 University Surgery Unit, Faculty of Medicine, University of Southampton, Southampton, UK; 4 Department of Child Health, Faculty of Medicine, University of Southampton, Southampton, UK

**Keywords:** Preterm infants, Feeding, Neonatology, Statistical methods, Nested case-control, Matching, Randomised controlled trial

## Abstract

**Background:**

A nested case-control study is an efficient design that can be embedded within an existing cohort study or randomised trial. It has a number of advantages compared to the conventional case-control design, and has the potential to answer important research questions using untapped prospectively collected data.

**Methods:**

We demonstrate the utility of the matched nested case-control design by applying it to a secondary analysis of the Abnormal Doppler Enteral Prescription Trial. We investigated the role of milk feed type and changes in milk feed type in the development of necrotising enterocolitis in a group of 398 high risk growth-restricted preterm infants.

**Results:**

Using matching, we were able to generate a comparable sample of controls selected from the same population as the cases. In contrast to the standard case-control design, exposure status was ascertained prior to the outcome event occurring and the comparison between the cases and matched controls could be made at the point at which the event occurred. This enabled us to reliably investigate the temporal relationship between feed type and necrotising enterocolitis.

**Conclusions:**

A matched nested case-control study can be used to identify credible associations in a secondary analysis of clinical trial data where the exposure of interest was not randomised, and has several advantages over a standard case-control design. This method offers the potential to make reliable inferences in scenarios where it would be unethical or impractical to perform a randomised clinical trial.

## Key messages

- A matched nested case-control design provides an efficient way to investigate causal relationships using untapped data from prospective cohort studies and randomised controlled trials
- This method has several advantages over a standard case-control design, particularly when studying time-dependent exposures on rare outcomes
- It offers the potential to make reliable inferences in scenarios where unethical or practical issues preclude the use of a randomised controlled trial

## Background

Randomised controlled trials (RCTs) are regarded as the gold standard in evidence based medicine, due to their prospective design and the minimisation of important sources of bias through the use of randomisation, allocation concealment and blinding. However, RCTs are not always appropriate due to ethical or practical issues, particularly when investigating risk factors for an outcome. If beliefs about the causal role of a risk factor are already embedded within a clinical community, based on concrete evidence or otherwise, then it is not possible to conduct an RCT due to lack of equipoise. It is often not feasible to randomise potential risk factors, for example, if they are biological or genetic or if there is a strong element of patient preference involved. In such scenarios, the main alternative is to conduct an observational study; either a prospective cohort study which can be complicated and costly, or a retrospective case-control study with methodological shortcomings.

The nested case-control study design employs case-control methodology within an established prospective cohort study [1]. It first emerged in the 1970–80s and was typically used when it was expensive or difficult to obtain data on a particular exposure for all members of the cohort; instead a subset of controls would be selected at random [2]. This method with the use of matching has been shown to be an efficient design that can be used to provide unbiased estimates of relative risk with considerable cost savings [3–5]. Cases who develop the outcome of interest at a given point in time are matched to a random subset of members of the cohort who have not experienced the outcome at that time. These controls may develop the outcome later and become a case themselves, and they may also act as a control for other cases [6, 7]. This approach has a number of advantages compared to the standard case-control design: (1) cases and controls are sampled from the same population, (2) exposures are measured prior to the outcome occurring, and (3) cases can be matched to controls at the time (e.g. age) of the outcome event.

More recently, the nested case-control design has been used within RCTs to investigate the causative role of risk factors in the development of trial outcomes [8–10]. In this paper we investigate the utility of the matched nested case-control design in a secondary analysis of the ADEPT: Abnormal Doppler Enteral Prescription Trial (ISRCTN87351483) data, to investigate the role of different types of milk feed (and changes in types of milk feed) in the development of necrotising enterocolitis. We illustrate the use of this methodology and explore issues relating to its implementation. We also discuss and appraise the value of this methodology in answering similar challenging research questions using clinical trial data more generally.

## Methods

### Adept

ADEPT: Abnormal Doppler Enteral Prescription Trial (ISRCTN87351483) was funded by Action Medical Research (SP4006) and investigated whether early (24–48 h after birth) or late (120–144 h after birth) introduction of milk feeds was a risk factor for necrotising enterocolitis (NEC) in a population of 404 infants born preterm and growth-restricted, following abnormal antenatal Doppler blood flow velocities [11]. Consent and randomisation occurred in the first 2 days after birth. There was no difference found in the incidence of NEC between the two groups, however there was interest in the association between feed type (formula/fortifier or exclusive mother/donor breast milk) and the development of NEC. Breast milk is one of few factors believed to reduce the risk of NEC that has been widely adopted into clinical practice, despite a paucity of high quality population based data [12, 13]. However, due to lack of equipoise it would not be ethical or feasible to conduct a trial randomising newborn infants to formula or breast milk.

With additional funding from Action Medical Research (GN2506), the authors used a matched nested case-control design to investigate the association between feed type and the development of severe NEC, defined as Bell’s staging Stage II or III [14], using detailed daily feed log data from the ADEPT trial. The feed type and quantity of feed was recorded daily until an infant had reached full feeds and had ceased parenteral nutrition, or until 28 days after birth, whichever was longest. Using this information, infants were classified according to the following predefined exposures:

1. Exposure to formula milk or fortifier in the first 14 days of life
2. Exposure to formula milk or fortifier in the first 28 days of life
3. Any prior exposure to formula milk or fortifier
4. Change in feed type (between formula, fortifier or breast milk) within the previous 7 days.

In the remainder of the methods section we discuss the challenges of conducting this analysis and practical issues encountered in applying the matched nested case-control methodology. In the results section we present data from different aspects of the analysis, to illustrate the utility of this approach in answering the research question.

### Cohort time axis

For the main trial analysis, time of randomisation was defined as time zero, which is the conventional approach given that events occurring prior to randomisation cannot be influenced by the intervention under investigation. However, for the nested case-control analysis, time zero was defined as day of delivery because age in days was considered easier to interpret, and also it was possible for an outcome event to occur prior to randomisation. Infants were followed up until their exit time, which was defined by the first occurrence of NEC, death or the last daily feed log record.

### Case definition

An infant was defined as a case at their first recorded incidence of severe NEC, defined as Bell’s staging Stage II or III [14]. Infants could only be included as a case once; subsequent episodes of NEC in the same infant were not counted. Once an infant had been identified as a case, they could not be included in any future risk sets for other cases, even if the NEC episode had been resolved.

### Risk set definition

**Fig. 1:**
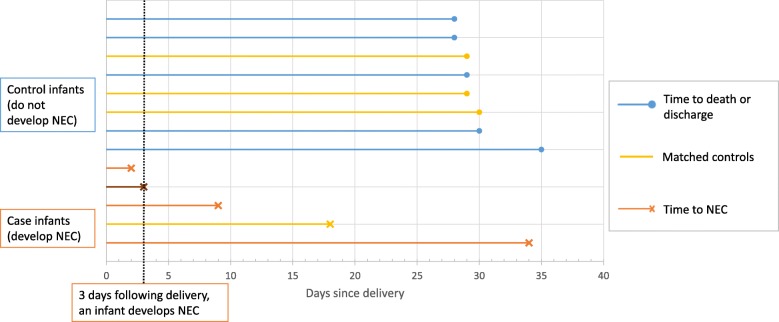
Schematic diagram illustrating the selection of controls from each risk set. Three days following delivery, an infant develops NEC. At this point, there are 11 infants left in the risk set. Four controls with the closest matching are selected, including one infant that becomes a future case on day 18

One of the major challenges was identifying an appropriate risk set from which controls could be sampled, whilst also allowing the analysis to incorporate the time dependent feed log data and adjust for known confounders. A diagnosis of NEC has a crucial impact on the subsequent feeding of an infant, therefore it was essential that the analysis only included exposure to non-breast milk feeds prior to the onset of NEC. A standard case-control analysis would have produced misleading results in this context, as infants would have been defined as a cases if they had experienced NEC prior to the end of the study period, regardless of the timing of the event in relation to exposure to non-breast milk. Using a matched nested case-control design allowed us to match an infant with a diagnosis of NEC (case) at a given point in time (days from delivery) to infants with similar characteristics (with respect to other important confounding factors), who had not experienced NEC at the failure time of the case. Figure 1 is a schematic diagram of this process. Each time an outcome event occurred (case), infants that were still at risk were eligible to be selected as a control (risk set). A matching algorithm was used to select a sample of controls with similar characteristics from this risk set. Infants selected as controls could go on to become a case themselves, and could also be included in the risk sets for other cases.

### Selection of matching factors

**Fig. 2:**
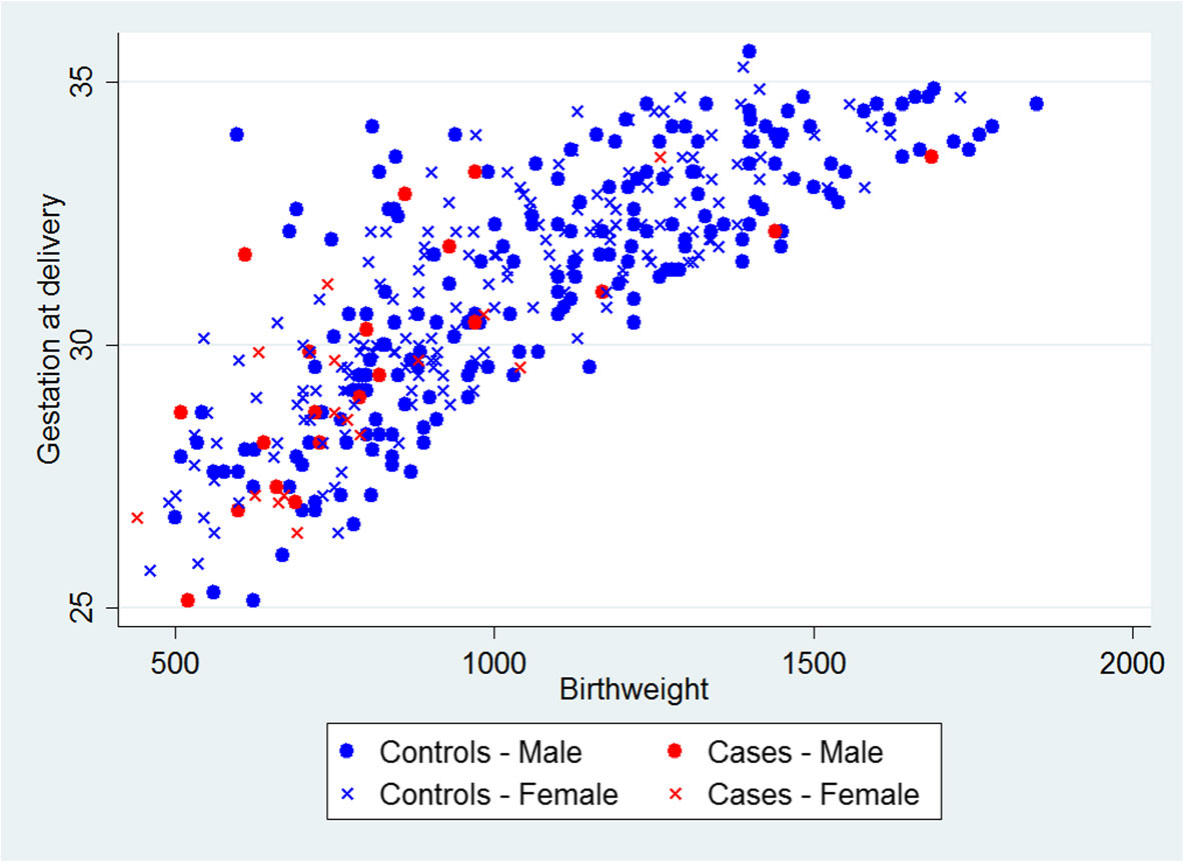
Scatterplot of birth weight versus gestational age for infants with NEC (cases) and those without (controls)

An important consideration was the appropriate selection of matching factors as well as identifying the optimum mechanism for matching. Sex, gestational age and birth weight were considered to be clear candidates for matching factors, as they are all associated with the development NEC. Gestational age and birth weight in particular are both likely to impact the infant’s feeding and thus their exposure to non-breast milk feeds. Both gestational age and birth weight were matched simultaneously, because of the strong collinearity between gestational age and birth weight, illustrated in Fig. 2. This was achieved by minimising the Mahalanobis distance from the case to prospective controls of the same sex [15]. That is, selecting the control closest in gestational age and birth weight to the case while taking into account the correlation between these characteristics.

Typically, treatment allocation would be incorporated as a matching factor since in a secondary analysis it is a nuisance factor imposed by the trial design, which should be accounted for. However, in this example, the ADEPT allocation is associated with likelihood of exposure, since it directly influences the feeding regime. For example, an infant randomised to receive early introduction of feeds is more likely to be exposed to non-breast milk feeds in the first 14 days (44%) than an infant randomised to late introduction of feeds (23%). The main trial results also demonstrated no evidence of association with the outcome (NEC) and therefore there was a concern about the potential for overmatching. Overmatching is caused by inappropriate selection of matching factors (i.e. factors which are not associated with the outcome of interest), which may harm the statistical efficiency of the analysis [16]. Therefore, we did not include the ADEPT allocation as a matching factor, but we conduct an unadjusted and adjusted analysis by trial arm, to examine its impact on the results.

### Selection of controls

Another important consideration was the method used to randomly select controls from each risk set for each case. This can be performed with or without replacement and including or excluding the case in the risk set. We chose the recommended option of sampling without replacement and excluding the case from the risk set, which produces the optimal unbiased estimate of relative risk, with greater statistical efficiency [17, 18]. However, infants could be included in multiple risk sets and be selected more than once as a control. We also included future cases of NEC as controls in earlier risk sets, as their exclusion can also lead to biased estimates of relative risk [19].

### Number of controls

In standard case-control studies it has been shown that there is little statistical efficiency gained from having more than four matched controls relative to each case [20, 21]. Using five controls is only 4% more efficient than using four, therefore there is no added benefit in using additional controls if a cost is attached, for example taking extra biological samples in a prospective cohort setting. However gains in statistical efficiency are possible by using more than four controls if the probability of exposure among controls is low (< 0.1) [4, 5]. Neither of these were issues for this particular analysis, as there were no additional costs involved in using more controls and prevalence of the defined exposures to non-breast milk was over 20% among infants without a diagnosis of NEC. However, there was a concern that including additional controls with increasing distance from the gestational age and birth weight of the case may undermine the matching algorithm. Also, increasing the number of controls sampled per case would lead to an increase in repeated sampling, resulting in larger number of duplicates present in the overall matched control population. This was a particular concern as control duplication was most likely to occur for infants with the lowest birth weight and gestational ages, from which there is a much smaller pool of control infants to sample from. This would have resulted in a small number of infants (with low birth weight and gestational age) being sampled multiple times and having disproportionate weighting in the matched control sample. Therefore, we limited the number of matched controls to four per case.

### Statistical analysis

The baseline characteristics of infants with NEC, the matched control group, and all infants with no diagnosis of NEC (non-cases) were compared. Numbers (with percentages) were presented for binary and categorical variables, and means (and standard deviations) or medians (with interquartile range and/or range) for continuous variables. Cases were matched to four controls with the same sex and smallest Mahalanobis distance based on gestational age and birth weight. Conditional logistic regression was used to calculate the odds ratio of developing NEC for cases compared matched controls for each predefined exposure with 95% confidence intervals. Unadjusted odds ratios were calculated, along with estimates adjusting for ADEPT allocation.

## Results

The results of the full analysis, including the application of this method to explore the relationship between feed type and other clinically relevant outcomes, are reported in a separate clinical paper (in preparation). Of the 404 infants randomised to ADEPT, 398 were included in this analysis (1 infant was randomised in error, 1 set of parents withdrew consent, 3 infants had no daily feed log data and for 1 infant the severity of NEC was unknown). There were 35 cases of severe NEC and 363 infants without a diagnosis of severe NEC (non-cases). Of the 140 matched controls randomly sampled from the risk set, 109 were unique, 31 were sampled more than once, and 8 had a subsequent diagnosis of severe NEC.

**Fig. 3:**
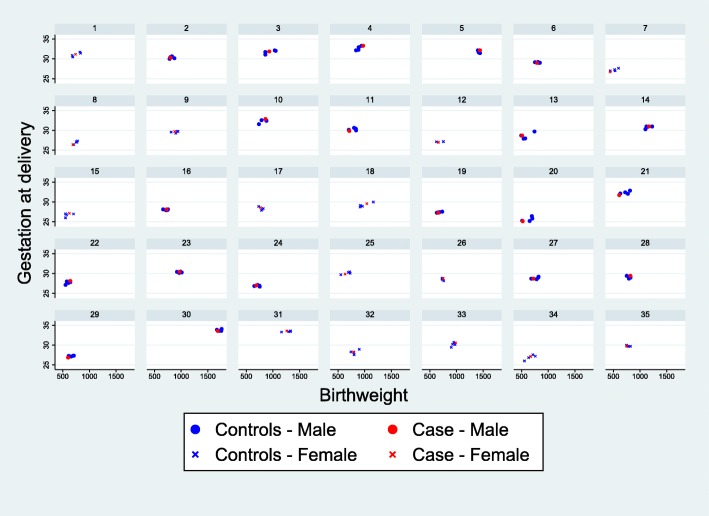
Scatterplots showing the matched cases and controls for each case of severe NEC. Each panel contains a separate case of NEC and the matched controls

The baseline characteristics of infants with severe NEC (cases) and their matched controls are shown in Table 1, alongside the characteristics of infants without a diagnosis of severe NEC (non-cases). The matching algorithm successfully produced a well matched collection of controls, based on the majority of these characteristics. There were, however, a slightly higher proportion of infants with the lowest birthweights (< 750 g) among the cases compared to the matched controls (49% vs 38%). The only other factors to show a noticeable difference between cases and matched controls are maternal hypertension (37% vs 49%) and ventilation at trial entry (6% vs 21%), neither of which have been previously identified as risk factors for NEC. Figure 3 shows scatter plots of birth weight and gestational age for the 35 individual cases of NEC and their matched controls, which provides a visual representation of the matching.

**Table 1:** Baseline characteristics of cases and matched controls for NEC (matching factors highlighted)

	Cases (severe NEC) (*n* = 35)	Matched controls (*n* = 140)^a^	Non-cases (*n* = 363)
Allocated to early arm, n (%)	17 (48.6)	74 (52.9)	182 (50.1)
Male sex, n (%)	20 (57.1)	80 (57.1)	193 (53.2)
Gestational age (weeks)			
Median (IQR)	29 (27 to 31)	29 (28 to 31)	32 (29 to 33)
< 29 weeks	16 (45.7)	61 (43.6)	69 (19.0)
> =29 weeks	19 (54.3)	79 (56.4)	294 (81.0)
Birth weight (grams)			
Median (IQR)	750 (660 to 931)	780 (695 to 890)	1030 (810 to 1280)
< 750 g	17 (48.6)	53 (37.9)	57 (15.7)
> =750 g	18 (51.4)	87 (62.1)	306 (84.3)
Multiple pregnancy, n (%)			
Twins	5 (14.3)	26 (18.6)	83 (22.9)
Triplets	0	3 (2.1)	6 (1.7)
Pregnancy induced hypertension, n (%)	13 (37.1)	69 (49.3)	145 (40.1)
Missing	0	0	1
Absent/reversed doppler abnormality, n(%)	35 (100.0)	138 (98.6)	347 (95.6)
Given antenatal steroids, n (%)	34 (97.1)	132 (95.7)	331 (91.4)
Missing	0	2	1
Onset of delivery, n (%)			
Spontaneous	2 (5.7)	2 (1.4)	7 (1.9)
Induced	0	1 (0.7)	4 (1.1)
Caesarean	33 (94.3)	137 (97.9)	352 (97.0)
Apgar score at 5 min			
Median (IQR)	9 (8 to 9)	9 (8 to 10)	9 (9 to 10)
Missing	2	2	7
Ventilated at trial entry, n (%)	2 (5.7)	30 (21.4)	45 (12.4)
CPAP^b^ at trial entry, n (%)	22 (62.9)	78 (55.7)	122 (33.6)
UAC^c^ in situ at trial entry, n (%)	11 (31.4)	35 (25.0)	44 (12.1)
UVC^d^ in situ at trial entry, n (%)	19 (54.3)	67 (48.6)	92 (25.4)
Missing	0	2	1
Any diagnosis of severe NEC^e^	35 (100.0)	8 (5.7)	0

^a^109 unique controls. Each case is matched to 4 controls with the same sex and the smallest distance in terms of the Mahalanobis distance based on gestational age and birth weight. For cases where the difference is the same, infants are selected at random. ^b^Continuous Positive Airway Pressure. ^c^Umbilical artery catheter. ^d^Umbilical venous catheter. ^e^Matched controls can include infants who develop severe necrotising enterocolitis at some time in the future

**Fig. 4:**
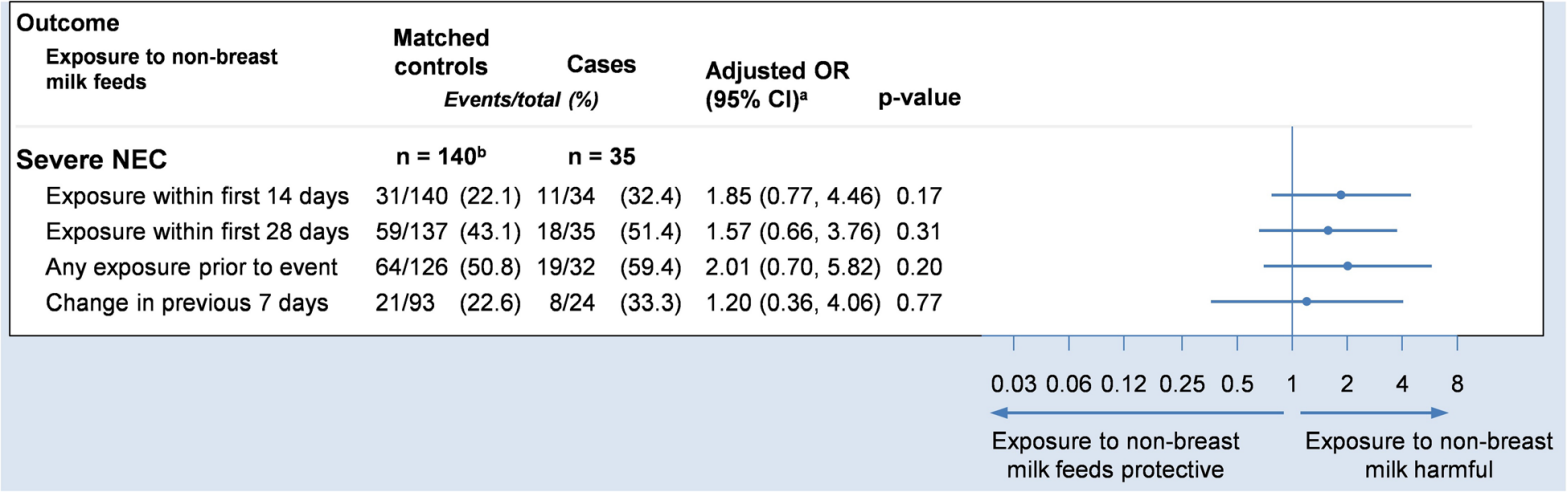
Forest plot showing the adjusted odds ratio comparing severe NEC to exposures. Odds ratios are adjusted for sex, gestational age and birthweight (via matching) and trial arm (via covariate adjustment). ^a^Odds ratio and 95% confidence interval. ^b^109 unique controls

The main results of the adjusted analysis are presented in Fig. 4. Unadjusted analyses are included in Table A1 in the supplementary material, alongside a post-hoc sensitivity analysis that additionally includes covariate adjustment for gestational age and birthweight. While the study did not identify any significant trends between feed-type and severe NEC the findings were consistent with the a priori hypothesis, that exposure to non-breast milk feeds is associated with an increased risk of NEC. In addition, the study identified some potential trends in the association of feed-type with other important outcomes, worthy of further investigation.

## Discussion

Employing a matched nested case-control design for this secondary analysis of clinical trial data overcame many of the limitations of a standard case-control analysis. We were able to select controls from the same population as the cases thus avoiding selection bias. Using matching, we were able to create a comparable sample of controls with respect to important clinical characteristics and confounding factors. This method allowed us to reliably investigate the temporal relationship between feed type and severe NEC since the exposure data was collected prospectively prior to the outcome occurring. We were also able to successfully investigate the relationship between feed type and several other important outcomes such as sepsis. A standard case-control analysis is typically based on recall or retrospective data collection once the outcome is known, which can introduce recall bias. If we had performed a simple comparison between cases and non-cases of NEC without taking into account the timing of the exposure, this would have produced misleading results. Another advantage of the matched nested case-control design was that we were able to match cases to controls at the time of the outcome event so that they were of comparable ages. The methodology is especially powerful when the timing of the exposure is of importance, particularly for time-dependent exposures such as the one studied here.

While the efficient use of existing trial data has a number of benefits, there are of course disadvantages to using data that were collected for another primary purpose. For instance, it is possible that such data are less robustly collected and checked. As a result, researchers may be more likely to encounter participants with either invalid or missing data.

For instance, the some of the additional feed log data collected in ADEPT were never intended to be used to answer clinical research questions, rather, their purpose was to monitor the adherence of participants to the intervention or provide added background information. In this study, it was necessary to make assumptions about missing data to fill small gaps in the daily feed logs. Researchers should take care that such assumptions are fully documented in the statistical analysis plan in advance and determined blinded to the outcome. Another option is to plan these sub-studies at the design phase, however, there needs to be a balance between the potential burden of additional data collection and having a streamlined trial that is able to answer the primary research question.

Another limitation of the methodology is that it is only possible to match on known confounders. This is in contrast to a randomised controlled trial, in which it is possible to balance on unknown and unmeasured baseline characteristics. As a consequence, particular care must be given to select important matching factors, but also to avoid overmatching.

The methodology allows for participants to be selected as controls multiple times, so there is the possibility that systematic duplication of a specific subset of participants (e.g. infants with a lower birthweight and smaller gestational age) could lead to a small number of participants disproportionately influencing the results. Within this study, we conducted sensitivity analyses with fewer controls, and were able to demonstrate that this had a minimal impact on the findings.

## Conclusions

We have demonstrated how a matched nested case-control design can be embedded within an RCT to identify credible associations in a secondary analysis of clinical trial data where the exposure of interest was not randomised. We planned this study after the clinical trial data had already been collected, but it could have been built in seamlessly as a SWAT (Study Within A Trial) during the trial design phase, to ensure that all relevant data were collected in advance with minimal effort. This method has several advantages over a standard case-control design and offers the potential to make reliable inferences in scenarios where unethical or practical issues preclude the use of an RCT. Moreover, because of the flexibility of the methodology in terms of the design and analysis, the matched nested case-control design could reasonably be applied to a wide range of challenging research questions. There is an abundance of high quality large prospective studies and clinical trials with well characterised cohorts, in which this methodology could be applied to investigate causal relationships, adding considerable value for money to the original studies.

## Supplementary information

**Additional file 1.** Table A1 Association between exposures and the development of Severe NEC. Each case is matched to 4 controls with the same sex and the smallest distance in terms of the Malhalanobis distance based on gestational age and birthweight.

## Data Availability

ADEPT trial data are available upon reasonable request, subject to the NPEU Data Sharing Policy.

## Abbreviations

ADEPT: Abnormal Doppler Enteral Prescription Trial
RCT: Randomised controlled trial
NEC: Necrotising enterocolitis
CPAP: Continuous positive airway pressure
UAC: Umbilical artery catheter
UVC: Umbilical venous catheter
SWAT: Study within a trial
