# Supplementary material for: Application of the matched nested case-control design to the secondary analysis of trial data
Source: BMC Med Res Methodol. 2020 May 14;20:117. doi: 10.1186/s12874-020-01007-w (PMC7227268; doi:10.1186/s12874-020-01007-w)
Supplement: Supplementary file 1 — Additional file 1. Table A1 Association between exposures and the development of Severe NEC. Each case is matched to 4 controls with the same sex and the smallest distance in terms of the Malhalanobis distance based on gestational age and birthweight. [file 12874_2020_1007_MOESM1_ESM.docx]

Table A1: Association between exposures and the development of Severe NEC. Each case is matched to 4 controls with the same sex and the smallest distance in terms of the Malhalanobis distance based on gestational age and birthweight.

|  | **Matched controls** | **Cases** | **Unadjusted OR (95% CI)** | **Adjusted^a^ OR (95% CI)** | **Further adjusted^b^ OR (95% CI)** |
| --- | --- | --- | --- | --- | --- |
|  |  |  |  |  |  |
| **Severe NEC** | **n = 140^c^** | **n = 35** |  |  |  |
| Exposure to formula milk or fortifier within first 14 days, n (%) | 31 (22.1) | 11 (32.4) | 1.79 (0.74, 4.32) | 1.85 (0.77, 4.46) | 1.75 (0.71, 4.30) |
| Missing | 0 | 1 |  |  |  |
|  |  |  |  |  |  |
| Exposure to formula milk or fortifier within first 28 days, n (%) | 59 (43.1) | 18 (51.4) | 1.53 (0.64, 3.62) | 1.57 (0.66, 3.76) | 1.50 (0.62, 3.63) |
| Missing | 3 | 0 |  |  |  |
|  |  |  |  |  |  |
| Any exposure to formula milk or fortifier prior to event, n(%) | 64 (50.8) | 19 (59.4) | 1.95 (0.68, 5.65) | 2.01 (0.70, 5.82) | 1.87 (0.64, 5.49) |
| Missing | 14 | 3 |  |  |  |
|  |  |  |  |  |  |
| Change in feed type in the seven days prior to event, n(%) | 21 (22.6) | 8 (33.3) | 1.33 (0.41, 4.37) | 1.20 (0.36, 4.06) | 1.11 (0.31, 3.95) |
| Missing | 47 | 11 |  |  |  |
| **^a^**Odds ratio adjusted for sex, gestational age and birthweight (via matching) and trial arm (via covariate adjustment)  **^b^**Odds ratio adjusted for sex, gestational age and birthweight (via matching) and trial arm, gestational age and birthweight (via covariate adjustment)  **^c^**109 unique controls | | | | | |
